# Chromosomal radiosensitivity in oncological and non-oncological patients with rheumatoid arthritis and connective tissue diseases

**DOI:** 10.1186/s13014-023-02291-8

**Published:** 2023-06-07

**Authors:** Dinah Rzepka, Hannah Schenker, Hans Geinitz, Elisabeth Silberberger, Dorothee Kaudewitz, Barbara Schuster, Lukas Kuhlmann, Miriam Schonath, Horacio Ayala Gaona, Bernhard Aschacher, Rainer Fietkau, Georg Schett, Luitpold Distel

**Affiliations:** 1 Department of Radiation Oncology, Universitätsklinikum Erlangen, Friedrich-Alexander-Universität Erlangen-Nürnberg (FAU), Universitätsstraße 27, 91054 Erlangen, Germany; 2 Comprehensive Cancer Center Erlangen-Europäische Metropolregion Nürnberg (CCC ER-EMN), Erlangen, Germany; 3 Department of Internal Medicine 3 - Rheumatology and Clinical Immunology, Friedrich-Alexander- Universität Erlangen-Nürnberg and Universitätsklinikum Erlangen, Erlangen, Germany; 4 Deutsches Zentrum Immuntherapie, Friedrich-Alexander-Universität Erlangen-Nürnberg and Universitätsklinikum Erlangen, Erlangen, Germany; 5 Department of Radiation Oncology, Ordensklinikum Linz Barmherzige Schwestern, Linz, Austria; 6 Department of Haematology, Oncology and Rheumatology, Universitätsklinikum Heidelberg, Heidelberg, Germany

**Keywords:** Radiation sensitivity, Radiosensitivity, Rheumatoid arthritis, Connective tissue disease, Collagen vascular disease, Radiotoxicity, Radiotherapy, Rheumatism, Chromosomal aberrations, Fluorescence in situ hybridisation

## Abstract

**Background:**

The risk of developing late radiotoxicity after radiotherapy in patients with high chromosomal radiosensitivity after radiotherapy could potentially be higher compared to the risk in patients with average radiosensitivity. In case of extremely high radiosensitivity, dose reduction may be appropriate. Some rheumatic diseases (RhD), including connective tissue diseases (CTDs) appear to be associated with higher radiosensitivity. The question arises as to whether patients with rheumatoid arthritis (RA) also generally have a higher radiosensitivity and whether certain parameters could indicate clues to high radiosensitivity in RA patients which would then need to be further assessed before radiotherapy.

**Methods:**

Radiosensitivity was determined in 136 oncological patients with RhD, 44 of whom were RA patients, and additionally in 34 non-oncological RA patients by three-colour fluorescence in situ hybridization (FiSH), in which lymphocyte chromosomes isolated from peripheral blood are analysed for their chromosomal aberrations of an unirradiated and an with 2 Gy irradiated blood sample. The chromosomal radiosensitivity was determined by the average number of breaks per metaphase. In addition, correlations between certain RA- or RhD-relevant disease parameters or clinical features such as the disease activity score 28 and radiosensitivity were assessed.

**Results:**

Some oncological patients with RhD, especially those with connective tissue diseases have significantly higher radiosensitivity compared with oncology patients without RhD. In contrast, the mean radiosensitivity of the oncological patients with RA and other RhD and the non-oncological RA did not differ. 14 of the 44 examined oncological RA-patients (31.8%) had a high radiosensitivity which is defined as ≥ 0.5 breaks per metaphase. No correlation of laboratory parameters with radiosensitivity could be established.

**Conclusions:**

It would be recommended to perform radiosensitivity testing in patients with connective tissue diseases in general. We did not find a higher radiosensitivity in RA patients. In the group of RA patients with an oncological disease, a higher percentage of patients showed higher radiosensitivity, although the average radiosensitivity was not high.

**Supplementary Information:**

The online version contains supplementary material available at 10.1186/s13014-023-02291-8.

## Background

While it is well established that different tumour entities differ in their resistance to radiation [1, 2], it is less known that individual patients also differ in their sensitivity to radiation [3, 4]. This has great significance in cancer therapy and is leading to increasingly individualized radiotherapy (RT) [4–8].

It is important to identify patients with high chromosomal radiosensitivity before RT is started [9]. These patients have a higher risk for developing adverse effects-such as late effects of RT as fibrosis or atrophia in the irradiation field with progression tendency-compared to those patients with a radiosensitivity that is within the average range [9–14]. However, sometimes the relationship between radiosensitivity and clinical late radiotoxicity is not entirely clear [12, 13, 15–18]. If the normal tissue is more sensitive to radiation, the toxicity of the RT will be greater and thus the probability of the occurrence of chronic toxicity will be greater [19]. Anyway, these are probabilities, so there is no certainty about the occurrence of undesirable therapeutic consequences [9, 19–21]. There is only a small number of patients with high radiosensitivity, for example, if 1000 patients are studied and the average patient has a risk probability of 1% per year due to a specific type of radiotherapy, approximately 50 patients will develop late toxicity within the next five years. If 10 of these 1000 patients have extremely high radiosensitivity and therefore, for example, risk of 5% per year from this particular therapy, only 2.5 radiosensitive patients will suffer from late toxicity even after five years, resulting in more adverse events in the average radiosensitive group. Additionally, side effects can occur years after treatment, and the modern therapies are unlikely to cause side effects. Therefore, it is difficult to detect the late effects and assign them to rare syndromes or diseases [19–21].

In the following, the term radiosensitivity refers to chromosomal radiosensitivity as measured ex vivo.The risk of late side effects in patients with high radiosensitivity can be diminished by reducing the radiation dose while at the same time there seem to be patients who have a higher resistance to ionizing radiation and who could benefit from dose escalation [3, 22, 23]. Since it is not possible to test all patients for their radiosensitivity using the complex chromosomal analyses, which can certainly predict high radiosensitivity [3, 21, 24–26], the targeted use of these tests is all the more important. At present, the radiation dose is generally chosen to be low to avoid exposing patients with high radiosensitivity to a high risk of serious late side effects [27, 28]. However, if the dose is too low, it may not be high enough to effectively inactivate all tumour cells [28]. By adjusting the radiation dose to the radiosensitivity of the individual patient, the chances of remission could be increased and the side effect profile could be minimized at the same time [28]. This work will primarily focus on the detection of radiosensitive patients, to reduce the incidence of late radiation effects after RT in general.

### Rheumatic diseases, connective tissue diseases and radiosensitivity

Independent of the interindividual variability of radiosensitivity, some diseases, syndromes, or genetic disorders seem to be associated with high radiosensitivity [29]. This is also postulated for rheumatic diseases (RhDs), including connective tissue diseases (CTDs) [30], such as systemic lupus erythematosus or systemic sclerosis/scleroderma [31–34]. As there are many contradictory statements in the literature on the topic of radiosensitivity and late toxicities of RT in patients with different RhDs [12, 31–33, 35, 36], the aim is to gain further insights into this field, with focus on rheumatoid arthritis (RA).

### Rheumatoid arthritis and radiosensitivity

RA is a chronic autoimmune systemic disease which represents the most common inflammatory rheumatic disease in the population [37]. Symmetrical, often relapsing polyarthritis with synovitis and joint destruction in the course is typical [38]. RA can also manifest extraarticular [39]. In industrialized countries, about up to 1% of the population is affected, and women are three times as often as men [40]. For the diagnosis and follow-up of RA, clinical examination and certain laboratory parameters are combined with imaging techniques to assess joint involvement, extension, and the presence of bone erosions [41, 42]. A distinction is made between seropositive and seronegative RA. In seropositive RA, rheumatoid factor and anti-citrullinated protein antibodies (anti-CCP) are elevated [43]. Autoantibodies against mutated citrullinated vimentin (anti-MCV) correlate with RA progression [44, 45]. The C-reactive protein (CRP) and the blood sedimentation rate (BSR) as non-specific inflammation parameters are also determined along with other laboratory values [41, 46]. In addition, the disease-activity score 28 (DAS-28), for example, should be measured regularly by a specialist to assess the current disease activity of RA. It consists of clinical parameters-such as the count and localization of painful, swollen joints, a patient self-assessment, and non-specific inflammation parameters [41, 47]. Treatment options for RA include medications such as systemic steroids, disease modifying antirheumatic drugs (DMARDs) like methotrexate (MTX), biologics, or intra-articular injections [48].

For the reasons mentioned above, it is important to find out whether there is a high risk for having a high radiosensitivity in oncological patients with RA (RA w/ CA).

In this work, we investigated whether patients with RA have higher radiosensitivity. We also examined whether laboratory parameters or clinical observations correlate with radiosensitivity.

## Methods (patients, material, and examination methods)

### Radiosensitivity measurement

Radiosensitivity was determined using a G0 three-colour fluorescence in situ hybridization assay (FiSH) as a radiosensitivity assay. In this assay, the chromosomes of lymphocytes isolated from peripheral blood were quantitatively analysed ex vivo for their chromosomal aberrations (breaks per metaphase, B/M) [26, 49]. The average B/M values of an unirradiated (0 Gy) and a 2.0 Gy irradiated blood sample were determined so that both the adequacy of the processing mechanisms of the radiation damage could be investigated and the value of the ex vivo radiosensitivity-hereafter sinply referred to simplistically only as B/M-could be determined.

### Patient selection

**Fig. 1:**
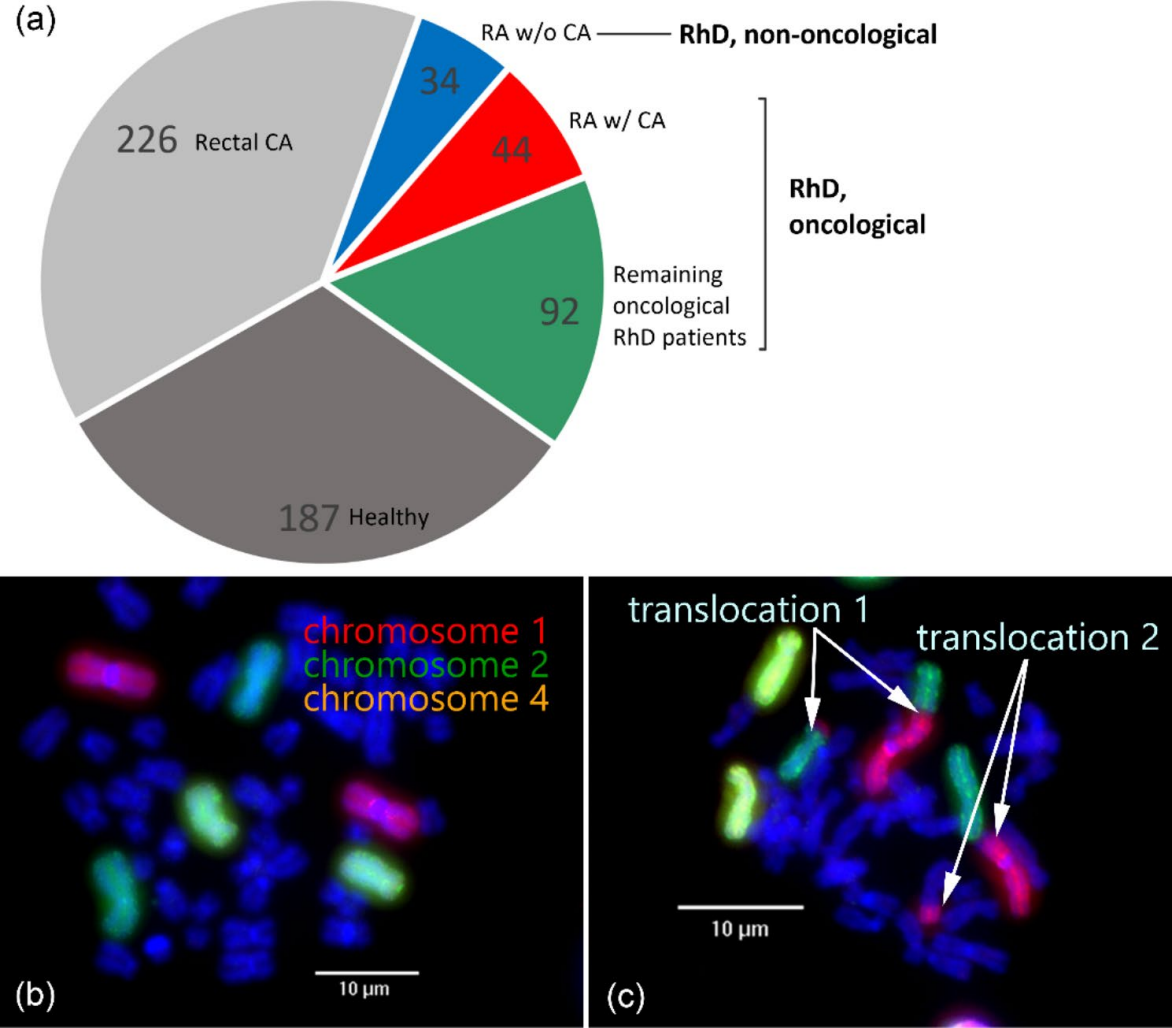
G0 three-colour fluorescence in situ hybridization assay (FiSH) as radiosensitivity assay. (**a**) The pie chart gives an overview of the patient groups that were used and the number of individuals in each of the groups. In addition to the healthy and rectal CA (rectal cancer) cohorts [50], there is also a cohort of patients with RhD (rheumatic disease), which can be subdivided into non-oncological (RA w/o CA - non-oncological patients with rheumatoid arthritis) and oncological patients. The latter includes the RA w/ CA cohort (oncological patients with rheumatoid arthritis). (**b**) Metaphase without chromosomal aberrations. (**c**) Metaphase with chromosomal aberrations. Chromosome #1 is translocated to chromosome #2-each chromosome is broken once (translocation 1). Chromosome #1 is translocated to another one coloured in blue-both are broken once each (translocation 2). In total, four breaks are counted in this metaphase

The radiosensitivity of 136 oncological patients with RhD who had not yet been irradiated or who had only received the first dose was determined. Of these, 44 oncological patients had RA (RA w/ CA) with a mean age of 63.8 years and a female proportion of 65.9% and the remaining patients had other RhDs. The most common oncological disease among the patients examined was breast cancer (Additional file 1). In addition, 34 non-oncological RA patients (RA w/o CA) with a mean age of 57.3 years and a female proportion of 73.5% were consecutively assessed for radiosensitivity. The other oncology patients with RhDs were suffering from psoriasis/psoriatic arthritis (n = 18), axial spondyloarthritis (n = 2), systemic lupus erythematosus (n = 15), systemic sclerosis/scleroderma (n = 9), dermatomyositis/polymyositis (n = 3), Sjögren’s syndrome (n = 4), polymyalgia rheumatica (n = 4), fibromyalgia syndrome (n = 6), sarcoidosis (n = 9), eosinophilic granulomatosis with polyangiitis (n = 2), granulomatosis with polyangiitis (n = 1), overlap syndrome (overlap, n = 2) and others (rheumatic symptoms, undifferentiated connective tissue disease or “rheumatism” as diagnosis, n = 17). In addition to the 170 patients with RhD, already existing samples from 187 healthy individuals and 226 patients with rectal cancer (rectal CA) without RhD were used for comparison (Fig. 1a) [50].

### Preparation and staining of chromosomes

As a standard for preparation and staining of chromosomes, we used a standardized protocol [51]. Approximately nine millilitres of heparinized blood was taken from each patient before irradiation. The blood from each person was divided and one-half was irradiated at room temperature with a 6 MeV Versa HD linear accelerator (Elekta, Stockholm, Sweden) at a dose of 2 Gy. A linear accelerator where patients are irradiated in clinical mode is used for blood irradiation. The dosimetry of the linac is performed according to the IEAE TRS 398 guideline. Therefore, the dose is controlled daily. The blood tubes are irradiated in an acrylic block 6 cm high, 14 cm wide, and 10 cm deep. The holes for the blood tubes are 2 cm from the surface. The dosimetry in this phantom at this position is performed by an ion chamber calibrated by an independent institute (licensed by the German national institute PTB).The other half was not irradiated and served as a background to show how many chromosomal aberrations were already present in the individual patient without irradiation. The lymphocytes were irradiated in the G0 phase. They were then stimulated with 6% phytohemagglutinin ((10576-015) Gibco (Life Technologies Cooperation, Grand Island, NY, USA)) and immediately incubated at 37 °C in RPMI-1640 medium (85%; Sigma-Aldrich, St. Louis, USA (R8758-500ml)) with 15% fetal calf serum (Sigma-Aldrich, St. Louis, USA (F7524-500ml)) and 1% Penicillin/Streptomycine ((15140-122)Gibco (Life Technologies Cooperation, Grand Island, NY, USA)). Lymphocytes with phytohemagglutinin were incubated uniformly for 48 h to obtain only those in the first mitosis after stimulation. Colcemid solution (final concentration 0.1 µg/mL; (15212-012) Gibco (Life Technologies Cooperation, Grand Island, NY, USA)) was used 3 ½ h before fixation to arrest the lymphocytes in metaphase. The chromosome suspension is dropped onto slides, and the air-dried slides are kept in 70% ethanol at − 20 °C for further use. Before performing the three-color FISH assay, a slide pretreatment is carried out to remove cell debris and improve the quality of the metaphase spreads. After fixing the metaphases on the slide, the samples were denaturated for 1 min 50 s at 72 °C and hybridized to the three largest chromosomes (#1, #2, and #4) to which the fluorescent dyes would later bind. In the last step, the chromosomes were stained accordingly with the fluorescent dyes-chromosomes #1 in red (Rhodamin), #2 in green (Fluorescein isothiocyanate), and #4 in yellow (Rhodamin and Fluorescein isothiocyanate) (Fig. 1b). A 0.4% FITC solution and a 1% antiavidin, 2.86% rhodamine solution were used.The deoxyribonucleic acid (DNA) was counterstained with 4′,6-diamidino-2-phenylindole (DAPI) so that the chromosomes could be recognized in the subsequent metaphase search [26, 52, 53].

### Image detection and analysis

Chromosomal aberrations were searched using a fluorescence microscope (Zeiss, Axioplan 2, Oberkochen, Germany) and Metasystem software (Metafer 4 V3.10.1, Altlussheim, Germany). The metaphases were first automatically searched in 10x magnification in DAPI filters. An image of each of these metaphases was taken at 63x magnification. These images were analysed using image analysis software (Biomas, Erlangen, Germany), where all chromosomal aberrations were scored according to the number of DNA breaks according to Savage and Simpson [52]. Chromosomal aberrations of one metaphase were scored as breaks per metaphase (B/M) (Fig. 1c). The background rates of the non-irradiated samples were subtracted from the rates of the samples irradiated with 2 Gy. The aim was to evaluate at least 200 metaphases per patient for the control (mean number of metaphases evaluated in the cohort RA w/o CA: n_metaphases_=214.4) and the blood sample irradiated with 2 Gy (n_metaphases_=180.8 in the cohort RA w/o CA); or as many as were available. For each metaphase, the stained chromosomes #1, #2, and #4 had to be checked for completeness and any chromosomal aberrations had to be noted. The mean value of the results gave the B/M value of the respective sample. The difference between these values of the unirradiated and the irradiated sample was used as a quantitative estimate of the radiosensitivity.

### Statistical analyses and methods

The SPSS Statistics 28 program (IBM, Armonk, New York, USA) was used for the analyses and statistical work. The statistical significance of the difference between two different cohorts was determined using the t-test for independent samples and Levene’s test (n > 30) and the non-parametric Man-Whitney U test (n < 30). The statistical significance of the correlations of specific values with the radiosensitivity was presented with the Spearman correlation (ρ, at n < 30) or the Pearson correlation (r, at n > 30). Charts, plots, and graphs were prepared using Excel (Microsoft Corporation, Redmond, Washington, USA) and Prism (GraphPad Software, San Diego, California, USA). It was always tested two-sided. The SD values in the tables represent parameters of the Gaussian normal distribution, adjusted to a specific mean.

### Comparison of radiosensitivity with laboratory values/ clinical parameters of the respective patients

Furthermore, a complementary method was to retrieve laboratory values from the hospital’s patient information system Soarian (Cerner, North Kansas City, USA). Metric laboratory values were correlated with radiosensitivity and dichotomous parameters were assessed for a difference between patient groups ≥ 0.5 B/M and < 0.5 B/M, respectively. The choice of the cut-off value of 0.5 B/M is based on several studies. A value of ≥ 0.5 B/M is therefore considered to be high radiosensitivity [54–57].

For regularly determined RA- or RhD-relevant disease parameters both the highest value reported in the patient record and the most recent value at the time of blood collection were used. The sex, the average age at blood collection, the length of pre-existing RA, IgG rheumatoid factor (RF), anti-CCPs, anti-MCV, non-specific inflammatory parameters (CRP and BSR), complement factors C3 and C4 and other values such as tumour necrosis factor α, interleukin-6, interleukin-2 receptor, single-stranded deoxyribonucleic acid-binding antibodies (anti-DNS), angiotensin-converting enzymes (ACE), ferritin, immunoglobulins A, E, G and M and the DAS-28 were examined as well as the presence of ANAs, ANCAs with cytoplasmic fluorescence pattern (c-ANCAs), ANCAs with perinuclear fluorescence pattern (p-ANCAs), erosions, adequately or inadequately controlled RA, recent MTX medication or recent systemic steroid medication.

## Results

### Radiosensitivity in oncological patients (RA w/ CA and patients with RhDs)

**Fig. 2:**
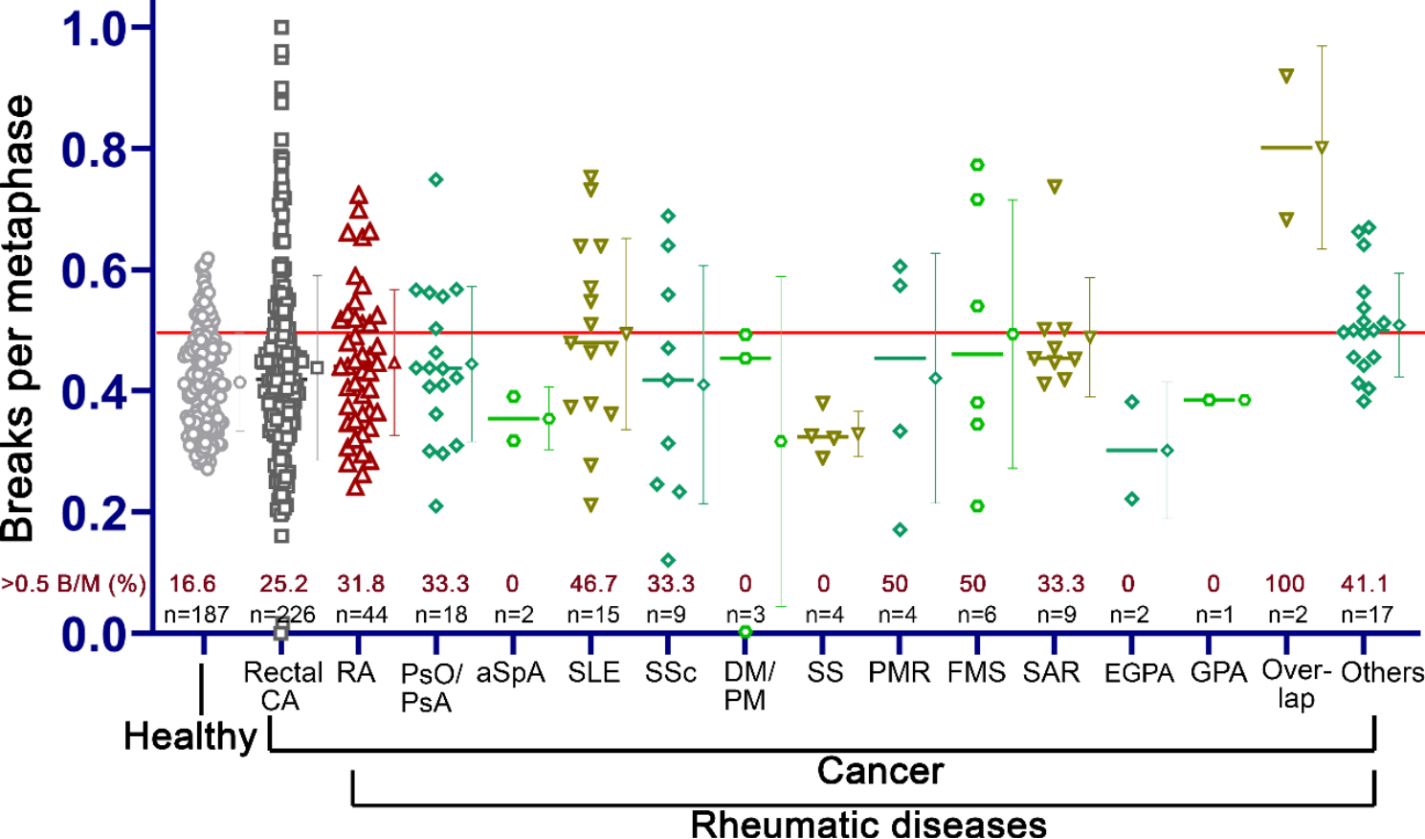
Radiosensitivity in healthy individuals, rectal CA patients [50] and oncological patients with rheumatic diseases. Each symbol represents the individual’s radiosensitivity measured with the three-colour FiSH. The horizontal red line at 0.5 B/M marks the values above which the radiosensitivity (breaks per metaphase, B/M) is considered higher. The horizontal line of the respective cohort represents the median, and the symbol to the right of it in the same colour marks the mean from which the standard deviation (SD) is displayed. Note the percentage of patients with ≥ 0.5 B/M. Legend: RA = rheumatoid arthritis, PsO/PsA = psoriasis/psoriatic arthritis, aSpA = axial spondyloarthropathy, SLE = systemic lupus erythematosus, SSc = systemic sclerosis/scleroderma, DM/PM = dermatomyositis/polymyositis, SS = Sjögren’s syndrome, PMR = polymyalgia rheumatica, FMS = fibromyalgia syndrome, SAR = sarcoidosis, EGPA = eosinophilic granulomatosis with polyangiitis, GPA = granulomatosis with polyangiitis, Overlap = overlap syndrome, Others = rheumatic symptoms or undifferentiated connective tissue disease or “rheumatism” as diagnosis.

The radiosensitivity of a total of 170 RhD patients was assessed by the three-colour fluorescence in situ hybridization assay (Fig. 1). They were compared with the radiosensitivity of 187 healthy individuals and with 226 patients with advanced rectal CA without RhD [50]. A threshold of 0.5 B/M is assumed for higher radiosensitivity. The 44 RA w/ CA patients had an average radiosensitivity of 0.45 B/M ± 0.12 B/M and were not different from the patients with rectal CA with 0.44 B/M. However, 31.8% of RA w/ CA patients had levels above 0.5 B/M while only 26.1% of rectal CA patients had elevated levels. 13 different non-RA RhD groups were investigated. Patients with overlap syndrome had a higher mean radiosensitivity, patients with Sjögren’s syndrome had a significant lower radiosensitivity compared to the reference cohort of patients with rectal CA. In the other RhD groups, no difference could be observed compared to the rectal CA cohort (Table 1; Fig. 2).

**Table 1:** Radiosensitivity in oncological patients with rheumatic diseases (RhD) compared to reference groups. Presentation of the radiosensitivity of various oncological patients with, which are also compared in tabular form with rectal CA (rectal cancer) patients [50] and non-oncological patients with rheumatoid arthritis (RA w/o CA). In addition to the mean radiosensitivity of the respective group, the mean age, the proportions of female and male patients are given. The significance value is given for the comparison with both rectal CA patients and oncological patients with rheumatoid arthritis (RA w/ CA patients). The number and proportion of patients with radiosensitivity > 0.5 B/M is also shown

	n	Age (years)	Sex female (%)/ male (%)	Radiosensitivity (Breaks per metaphase) mean ± SD	p (compared to patients with rectal CA)	p (compared to patients with RA w/ CA)	Patients ≥ 0.5 B/M n (%)
**Healthy**	187	---	---	0.42 ± 0.08	**0.049**	---	31 (16.6)
**Rectal CA**	226	50.6	96(42.7)/130(57.3)	0.44 ± 0.15	---	---	59 (26.1)
**RA w/o CA**	34	57.3	25(73.5)/19(26.5)	0.42 ± 0.07	---	---	5 (14.7)
**RA w/ CA**	44	63.8	32(72.7)/12(27.3)	0.45 ± 0.12	0.700	---	14 (31.8)
* RA w/ CA with another RhD	5	68.2	2(40)/3(60)	0.41 ± 0.07	0.715	---	1 (20)
* RA w/ CA without another RhD	39	63.2	30(76.9)/9(23.1)	0.45 ± 0.13	0.403	---	13 (33.3)
**Psoriasis/Psoriatic arthritis**	18	59.8	14(77.8)/4(22.2)	0.45 ± 0.12	0.611	0.981	6 (33.3)
Only Psoriasis	15	60.5	11(73.3)/4(26.7)	0.45 ± 0.14	0.656	0.979	6 (40)
Only Psoriatic arthritis	3	56.3	3(100)/0(0)	0.44	0.792	0.896	0 (0)
**Axial spondyloarthitis**	2	84.5	1(50)/1(50)	0.35 ± 0.04	0.267	0.215	0 (0)
**Systemic lupus erythematosus**	15	59.4	11(73.3)/4(26.7)	0.49 ± 0.15	0.110	0.261	7 (46.7)
**Systemic sclerosis/scleroderma**	9	62.9	6(66.7)/3(33.3)	0.41 ± 0.18	0.689	0.522	3 (33.3)
**Dermatomyositis/polymyositis**	3	45.7	0(0)/3(100)	0.32 ± 0.22	0.755	0.632	0 (0)
**Sjögren’s syndrome**	4	61.0	4(100)/0(0)	0.33 ± 0.03	**0.050**	**0.040**	0 (0)
**Polymyalgia rheumatica**	4	69.3	1(25)/3(75)	0.42 ± 0.18	0.982	0.896	2 (50)
**Fibromyalgia syndrome**	6	61.8	6(100)/0(0)	0.49 ± 0.20	0.622	0.654	3 (50)
**Sarcoidosis**	9	63.0	4(44.4)/5(55.6)	0.49 ± 0.09	0.114	0.297	3 (33.3)
**Eosinophilic granulomatosis with polyangiitis**	2	63.0	2(100)/0(0)	0.30 ± 0.08	0.154	0.118	0 (0)
**Granulomatosis with polyangiitis**	1	75.0	0(0)/1(100)	0.39	0.647	0.590	0 (0)
**Overlap syndrome (without RA)** Overlap 1: Systemic lupus erythematosus + Sjögren’s syndrome Overlap 2: Sarcoidosis + Sjögren’s syndrome	2	55.0	2(100)/0(0)	0.80 ± 0.12 (Overlap 1: 0.92 Overlap 2: 0.68)	**0.027**	**0.024**	2 (100)
Others	17	65.4	15(88.2)/2(11.8)	0.51 ± 0.08	**0.004**	**0.039**	9 (52.9)

The statistical comparison of RA patients with healthy donors is presented in Additional file 3.

* Difference between RA w/CA with another rheumatic disease (a.e. Sjögren’s syndrome or systemic lupus erythematosus, not fibromyalgia syndrome) and RA w/CA without another rheumatic disease: p = 0.43.

Legend: RA = rheumatoid arthritis,, others = rheumatic symptoms or undifferentiated connective tissue disease or “rheumatism” as diagnosis

### Radiosensitivity in patients with RA (RA w/ CA and RA w/o CA)

**Fig. 3:**
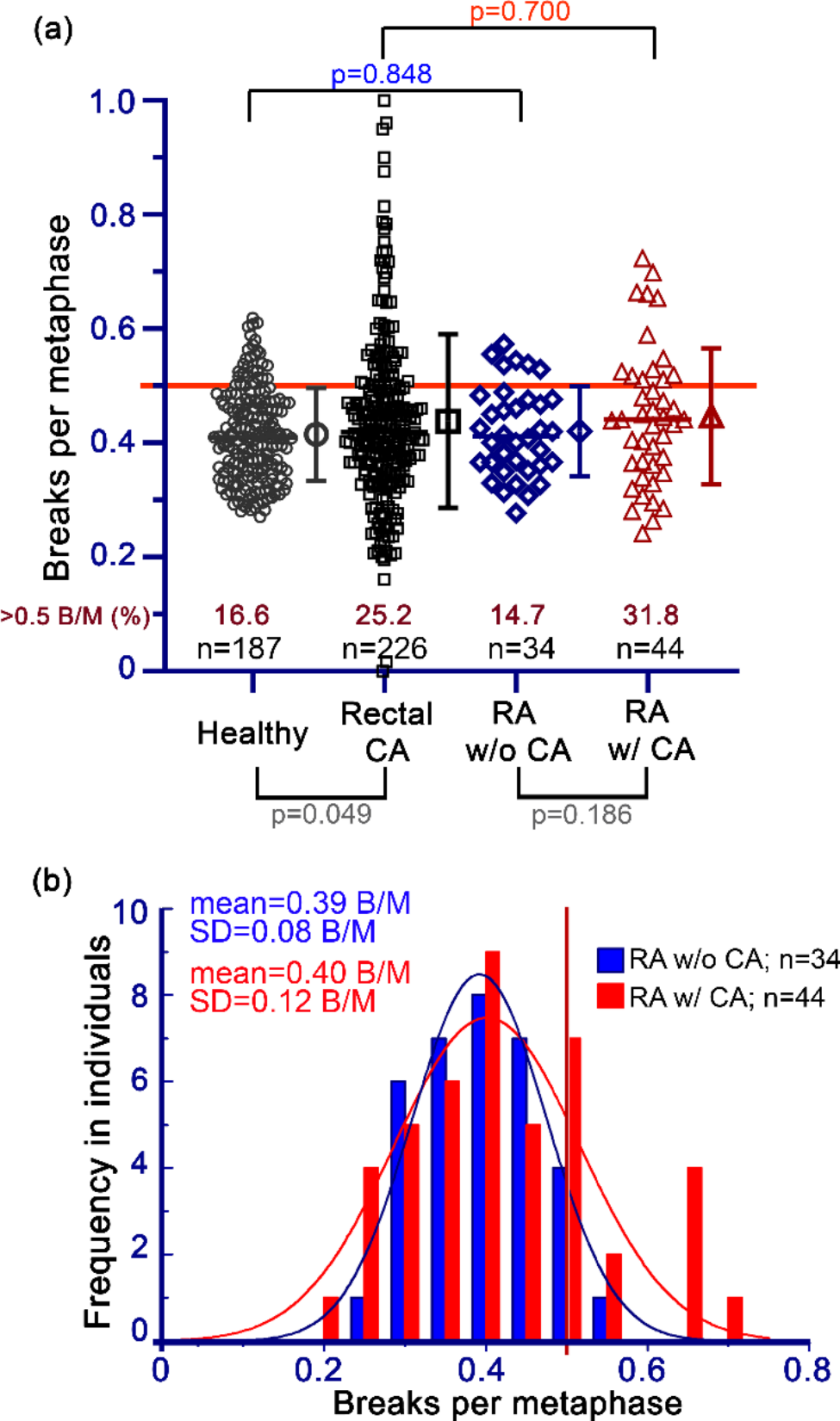
Radiosensitivity in non-oncological and oncological patients with rheumatoid arthritis. (**a**) Each symbol represents the radiosensitivity (breaks per metaphase, B/M) with the 3-colour FiSH. Comparison of the measured radiosensitivity of the patients with RA w/ CA (oncological patients with rheumatoid arthritis) and RA w/o CA (non-oncological patients with rheumatoid arthritis) cohorts with those of healthy individuals and with rectal cancer (rectal CA) patients [50]. In addition, the two comparison cohorts (healthy and rectal CA) and the two cohorts RA with CA and RA without CA were compared. Note the percentage of patients with ≥ 0.5 B/M. The horizontal line of the respective cohort represents the median, the symbol to the right of it in the same colour marks the mean from which the standard deviation (SD) is displayed. (**b**) The mean and the SD of the Gaussian normal distribution are calculated from the values of RA w/o and w/ CA. A vertical line at 0.5 B/M separates high and average radiosensitivity

The 44 RA w/ CA patients were compared with 34 RA w/o CA patients (Additional file 2). The radiosensitivity of the investigated RA w/o CA patients was 0.42 B/M, which was not different from the healthy cohort. There were five patients with radiosensitivity greater than 0.5 B/M and of these, one patient had a radiosensitivity greater than 0.55 B/M. The radiosensitivity of RA w/ CA patients is not different (p = 0.186) from the cohort of RA w/o CA patients, while the two comparison cohorts of healthy individuals (non-oncological) and rectal CA patients (oncological) were different (p = 0.049) (Fig. 3a) [58, 59]. It is noticeable that the standard deviation (SD) in RA w/ CA seems to be larger than that of RA w/o CA, which is also the case in rectal CA patients who have a higher SD than the healthy cohort. When comparing the respective radiosensitivity within the RA w/ CA and RA w/o CA cohorts, the Pearson chi-square test yielded a significance value of 0.415. Thus, the distributions of the B/M values are not significantly different (Fig. 3b).

### Correlation of radiosensitivity with RA-related disesase-parameters in RA w/o CA patients

**Fig. 4:**
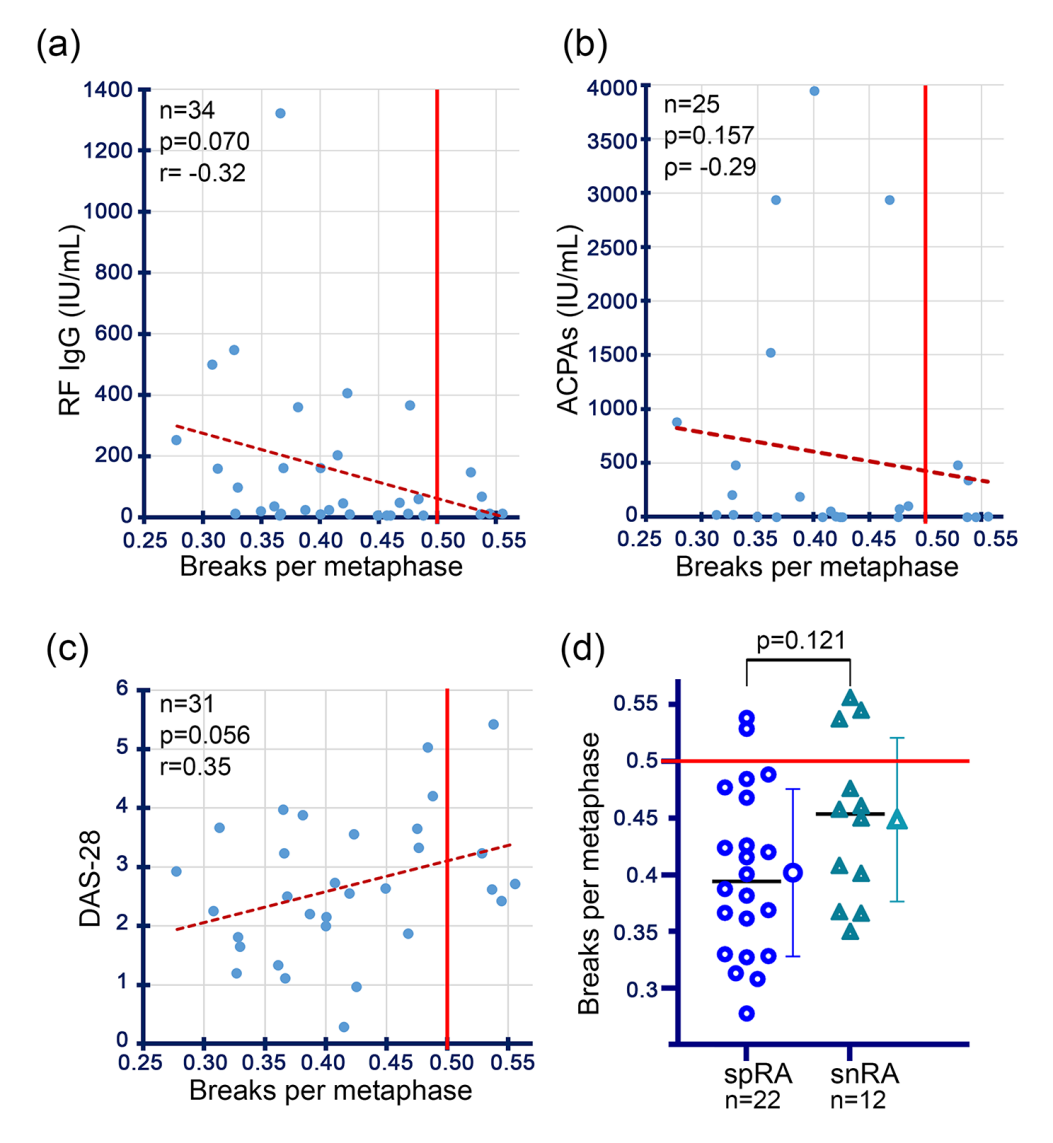
Correlation of laboratory values and disease activity in non-oncological patients with rheumatoid arthritis with their radiosensitivity. (**a**) Correlation of radiosensitivity (breaks per metaphase, B/M) and IgG rheumatoid factor (RF, highest value). (**b**) Correlation of B/M and anti-citrullinated protein antibodies (ACPAs, highest value). (**c**). Correlation of B/M and disease activity score 28 (DAS-28, most recent value). (**d**) Comparison of B/M of patients with seropositive rheumatoid arthritis (spRA) with those with seronegative rheumatoid arthritis (snRA).

No significant correlations between different RA-related disease-parameters and the radiosensitivity were found in the study of RA w/o CA patients. Furthermore, the distribution of radiosensitivity in patients with seropositive RA w/o CA patients did not differ from that in seronegative RA w/o CA patients. Similarly, the proportion of seropositive RA did not differ from seronegative RA in patients with radiosensitivity of ≥ 0.5 B/M and < 0.5 B/M, respectively (Fig. 4d) (Additional file 3). Patients with an additional RhD beside the RA did not differ in their average radiosensitivity from those without an additional RhD. The most recent DAS-28 shows a tendency to correlate with radiosensitivity (p = 0.056) (Fig. 4c). The proportion of patients with inadequately controlled RA, bone erosions, current systemic steroid therapy, and current MTX therapy did not differ in the < 0.5 B/M and ≥ 0.5 B/M patient groups (Fig. 4i-l). (Additional file 4, Additional file 5)

## Discussion

Previous studies suggest a high risk of late toxicities after RT in oncologic RhD patients; In some reports, the data are uncertain [19, 31, 32, 35, 60], particularly with regard to which RhDs they are referring to [31–33, 61, 62]. The radiosensitivity in some patients with connective tissue diseases differs from the rectal CA reference group without RhD. These patients could benefit from radiosensitivity testing. If the measured radiosensitivity in these patients is greater than 0.55 B/M, it may be possible to reduce the daily fractionation dose to reduce the risk of adverse therapeutic effects [26]. Already from 0.5 B/M, radiosensitivity is considered high, meaning that patients are more likely to be atrisk of undesirable late adverse effects than those with average radiosensitivity [26, 58, 59].

Furthermore, given the conflicting data, it seems reasonable to prioritise especially patients with RhD, in whom high radiosensitivity or a high risk of adverse effects from radiotherapy are more frequently reported in the literature, when testing capacity for chromosomal analysis is scarce, even if no clear recommendations can be made in this regard. It appears that oncological patients with systemic sclerosis/scleroderma in particular are at high risk of late radiotoxicity [31, 32, 36, 63–67] as are patients with systemic lupus erythematosus, although the data are inconclusive [14, 31, 32, 36, 63, 68–72]. This is partially consistent with the results of our in vitro radiosensitivity measurements performed for systemic lupus erythematosus and systemic sclerosis/scleroderma. The percentage of patients with systemic lupus erythematosus and systemic sclerosis/scleroderma with high mean radiosensitivity is higher than in the reference group of patients with rectal CA, although the difference is not statistically significant, possibly due to the small sample size. It should be noted, however, that the high radiosensitivity is not directly linked to a generally high incidence of radiation toxicity [12, 13, 15]. There is only a limited amount of information in the literature on oncological patients with psoriasis/psoriatic arthritis, axial spondyloarthritis, dermatomyositis/polymyositis, Sjögren’s syndrome, polymyalgia rheumatica, sarcoidosis, granulomatosis with polyangiitis, and eosinophilic granulomatosis with polyangiitis, so that no clear recommendation can be made regarding regular radiosensitivity testing [31, 32, 73, 74]. There are insufficient data in the literature on the radiosensitivity of patients with Sjögren’s syndrome to support or refute the lower mean radiosensitivity found in our investigations. Whether patients with sarcoidosis tend to have an increased radiosensitivity compared to oncological patients without RhD also needs to be determined by further research.

The finding in the chromosomal analyses that the radiosensitivity of the RA w/ CA (and RA w/o CA) cohort is not higher compared to the respective reference cohort only partially coincides with clinical experience with late toxicities in the literature [31–33, 61, 62]. For example, a recent meta-analysis found a significantly higher incidence of moderate late toxicities in a total of 137 oncological RA patients compared with the reference cohort without RA [31]. Given the mean radiosensitivity found in RA w/ CA patients, which is not significantly higher than in the rectal CA cohort, we would not recommend routine radiosensitivity testing prior to RT unless there are no other risk factors for high radiosensitivity in the individual patient.

Further insights could be gained by examining a larger patient cohort RA w/ CA. In summary, it should be noted that the the literature reports a high incidence of adverse effects from RT and that some patients in this RA w/ CA cohort also had a high radiosensitivity-however, this is also the case in oncological non-RhD patients, such as rectal CA patients. In addition to studies dealing with the ex vivo detection of radiosensitivity, it should be discussed whether it might be due to the design of some studies may lead to discrepancies between RA patients and other RhD patients in assumptions about their risk of developing late toxicities, as patients may not be followed long enough after RT to capture important late effects.

At the current stage of research, it is not realistic to expect that a stand-alone test of chromosomal radiosensitivity based on ex vivo markers that will reliably predict toxic effects- particularly late effects of RT-and lead to an adjustment of the patient’s treatment regimen [18]. The assumption of some intra-individual variability of chromosomal aberrations in radiosensitivity measurements supports the notion that conclusions about further therapeutic procedures should not be drawn from a single ex vivo test alone [75]. Therefore, our findings should be treated with caution and need to be supported by further studies, such as prospective observation of large patient populations with toxic effects and risk analyses for the development of different degrees of radiotoxicity [18].

Increasing the dose at which patients’ blood samples are irradiated (e.g. 5 Gy instead of 2 Gy) or using multiple doses (e.g. 0 Gy, 2 Gy, 5 Gy) may be a way to make the correlation between chromosomal and clinical radiosensitivity more apparent [18]. We did not find a correlation between RA- or RhD-relevant disease parameters in RA patients and increased radiosensitivity. However, certain parameters of the RA w/o CA cohort give the impression that there may be a tendency to correlate with radiosensitivity. It is conceivable that there is a tendency for the DAS-28 to correlate positively with radiosensitivity (Fig. 4g). It would be interesting to examine larger cohorts to see out if there is a significant correlation between disease activity and radiosensitivity. Another question that could be addressed is whether radiosensitivity changes with inflammatory activity-for example, whether it also correlates intra-individually with radiosensitivity at different time points. It has already been described in the literature that the activity of the underlying disease or a high titre of certain antibodies-for example in systemic sclerosis/scleroderma or systemic lupus erythematosus -could be another factor influencing radiosensitivity [14, 60]. Comparable studies have not yet been found in the literature.

### Limitations

In patients with high disease activity, there is a greater risk that the sample will not be sufficiently stimulable if high doses of systemic steroids (prednisolone > 20 mg/day) have been administered at the time of blood collection, resulting in too few or no evaluable metaphases at the end. Bias may be introduced by the possible limited testing of patients with acute flares requiring high systemic steroid doses.

Fewer than 200 evaluable metaphases were detected at 0 Gy in seven RA w/o CA patients and at 2 Gy in 18 patients in the RA w/o CA cohort. This may lead to small inaccuracies in the radiosensitivity measurements.

There is a correlation between radiosensitivity and the risk of developing late toxicities after RT, but it is never possible to say with certainty which patient will develop such late sequelae, so conclusions cannot be drawn directly from the ex vivo studies about the clinical development of radiotoxicity. In summary, the data has shown that radiosensitivity testing maybe may be more appropriate in certain RhD. By comparing various RA- or RhD-relevant disease parameters with radiosensitivity in RA patients, a further approach was taken to explore the complex issue of radiosensitivity in RhD patients. Inconsistencies within the literature and between the literature and our own data, which are ubiquitous in this field of research, were highlighted. In order to gain further insight into this area of research, it is necessary to investigate larger cohorts of RhD patients for radiosensitivity and to complement the studies by clinically recording the development of late complications in these patients.

## Conclusion

In order to to increase the validity of chromosomal radiosensitivity measurements, patients with RhD should continue to be tested for radiosensitivity in trials, e.g. using different methods of ex vivo biodosimetric determinations and in combination with the clinical outcome [18]. The aim would be to gain further insight into their risk of high radiosensitivity as a risk factor for late sequelae of therapy and to better assess the urgency and usefulness of chromosomal analysis in these patient groups. In this context, recent findings on the clinical impact of RT on late toxicities in the current literature should be taken into account.

Finally, both the determined average mean radiosensitivity of patients with RA w/CA and the parameters of RA w/o CA listed above should only be considered as small indications and should be considered in the overall picture, especially in combination with other present or absent risk factors of the individual patient. Like the findings in RhDs, they do not currently allow any reliable conclusions to be drawn about the radiosensitivity of individual patients or the expected risk of late radiotoxicity, nordo they allow RT to be adjusted, such as reducing the daily radiation dose without prior chromosomal analysis. The studies suggest that it is plausible to test patients with certain RhD for radiosensitivity prior to irradiation. Due to the lack of significance, it is not possible to make a general recommendation that radiosensitivity testing should be performed in patients with RA. Patients with RhD should be well monitored and educated during RT [76], but if in doubt, they should probably not be denied RT because of their underlying disease according to modern standards [72], especially if no alternative therapy is available, as the more severe radiotoxicity seems to be more common in RhD patients, but this is still rare overall [31]. In the future, studies focusing on the correlation between the in vitro measured radiosensitivity of oncological RhD patients and the severity of their clinical side effects, as well as investigations identifying any rheumatological indicator parameters that may indicate a high radiosensitivity, could contribute to a more targeted use of chromosomal analysis and an individualised RT and cancer therapy for the individual patient.

## Electronic supplementary material

Below is the link to the electronic supplementary material.

Additional file 1:
Title: Tumour entities of oncological patients with rheumatoid arthritis and other rheumatic diseases. Description: Frequency of occurrence of different tumour entities in the RA w/ CA cohort (oncological patients with rheumatoid arthritis) and in the other investigated oncological patients with rheumatic diseases (RhD) other than RA (rheumatoid arthritis). The column “RhDs total” combines these two subgroups. Legend: CA = cancer/carcinoma, malignant tumour disease.

Additional file 2:
Title: Comparison of the oncological and non-oncological patients with rheumatoid arthritis. Description: Comparison of the radiosensitivity (breaks per metaphase, B/M) and the measured unirradiated blood sample (0 Gy) and the irradiated blood sample (2 Gy) values. Legend: RA w/ CA = oncological patients with rheumatoid arthritis, RA w/o CA = non-oncological patients with rheumatoid arthritis.

Additional file 3:
Title: Comparison of subgroups of non-oncological patients with rheumatoid arthritis. Description: Comparison of the radiosensitivity (breaks per metaphase, B/M) in non-oncological patients with rheumatoid arthritis (RA w/o CA) with and without further rheumatic disease (RhD) and comparison of B/M in patients with seropositive RA (spRA) with those with seronegative (snRA) in each case with each other and with healthy individuals.

Additional file 4:
Title: Overview of laboratory values in non-oncological patients with rheumatoid arthritis. Description: Tabulation of the mean values and standard deviation (SD) of certain values and their correlation with radiosensitivity (breaks per metaphase, B/M). Note: of some values, both the most recent and the highest values listed are given. Legend: RF = IgG rheumatoid factor, CCP = anti-cyclic citrullinated peptide, ACPAs = anti-citrullinated protein antibodies, Anti-MCV = Autoantibodies against mutated citrullinated vimentin, BSR = blood sedimentation rate, CRP = C-reactive protein, Anti-DNS = anti-DNS-antibodies, DAS-28 = disease-activity-score 28.

Additional file 5:
Title: Comparison of values in non-oncological rheumatoid arthritis with increased and non-increased radiosensitivity. Description: Tabular overview of different dichotomous values of non-oncological patients with rheumatoid arthritis (RA w/o CA) in patients with an radiosensitivity (breaks per metaphase, B/M) of < 0.5 B/M or ≥ 0.5 B/M. Note: of some values, both the most recent and the highest values listed are given. Legend: ANA = anti-nuclear antibodies; ANCA = anti-citrullinated protein antibodies; MTX = methotrexate.

## Data Availability

The datasets generated and/or analysed during the current study are not publicly available due but are available from the corresponding author on reasonable request.

## List of abbreviations

#: Number, e.g. chromosome #1
%: Percent
°C: Degree Celsius
ρ: Spearman’s Rho, Spearman’s correlation coefficient
0 Gy: Unirradiated blood sample
2 Gy: With 2 Gy irradiated blood sample
ACPAs: Anti-citrullinated protein antibodies
ANA: Anti-nuclear antibodies
ANCAs: Anti-neutrophil cytoplasmic antibodies
anti-DNS: Single-stranded desoxiribonucleic acid-binding antibodies
anti-MCV: Autoantibodies against mutated citrullinated vimentin
aSpA: Axial spondyloarthritis
B/M: Breaks per metaphase, chromosomal radiosensitivity
BSR: Blood sedimentation rate
c-ANCA: Anti-neutrophil cytoplasmic antibodies with cytoplasmic fluorescence pattern
CA: Cancer/carcinoma, malignant tumour disease
CCPs: Anti-cyclic citrullinated peptides
CRP: C-reactive protein
CTDs: Connective tissue diseases
DAPI: 4′,6-diamidino-2-phenylindole
DAS-28: Disease-activity-score 28
DM/PM: Dermatomyositis/polymyositis
DNA: Deoxyribonucleic acid
e.g.: For example
EGPA: Eosinophilic granulomatosis with polyangiitis
FiSH: Fluorescence in situ hybridization assay
FMS: Fibromyalgia syndrome
G0: Resting phase of the cell cycle
GPA: Granulomatosis with polyangiitis
Gy: Gray
h: Hours
IU/mL: International unit per millilitre
mg/dl: Milligram per decilitre
mg/l: Milligram per litre
mm/h: Millimetre per hour
MTX: Methotrexate
n: Number of patients
ng/mL: Nanogram per millilitre
p-ANCA: Anti-neutrophil cytoplasmic antibodies with perinuclear fluorescence pattern
p: Significance value
pg/mL: Picogram per millilitre
PMR: Polymyalgia rheumatica
PsA: Psoriatic arthritis
PsO: Psoriasis
r: Pearson’ correlation coefficient
RA: Rheumatoid arthritis
RA w/ CA: oncological patients with rheumatoid arthritis
RA w/o CA: non-oncological patients with rheumatoid arthritis
RF: IgG rheumatoid factor
RhDs: Rheumatic diseases
RT: Radiotherapy
RU/mL: Relative units per millilitre
SAR: Sarcoidosis
SD: Standard deviation
SLE: Systemic lupus erythematosus
snRA: Seronegative rheumatoid arthritis
spRA: Seropositive rheumatoid arthritis
SS: Sjögren’s syndrome
SSc: Systemic sclerosis/scleroderma
w/: With
w/o: Without
